# Personal recovery among people with opioid use disorder during treatment with extended-release naltrexone

**DOI:** 10.1016/j.heliyon.2023.e17516

**Published:** 2023-07-05

**Authors:** Anne Marciuch, Bente Birkeland, Jūratė Šaltytė Benth, Kristin Klemmetsby Solli, Lars Tanum, Ida Mathisen, Bente Weimand

**Affiliations:** a Department of Research and Development in Mental Health, Akershus University Hospital, Loerenskog, Norway; b Faculty of Medicine, Institute of Clinical Medicine, University of Oslo, Oslo, Norway; c Department of Psychosocial Health, University of Agder, Kristiansand, Norway; d Institute of Clinical Medicine, Campus Ahus, University of Oslo, Norway; e Health Services Research Unit, Akershus University Hospital, Lørenskog, Norway; f Norwegian Centre for Addiction Research, University of Oslo, Norway; g Vestfold Hospital Trust, Toensberg, Norway; h Faculty for Health Science, Oslo Metropolitan University, Oslo, Norway; i Faculty of Health and Social Sciences, University of South-Eastern Norway, Norway; j Department of Health, Social and Welfare Studies, University of South-Eastern Norway, Drammen, Norway

**Keywords:** Personal recovery, QPR, Extended-release naltrexone, XR-NTX, Opioid use disorder, Opioids

## Abstract

**Background and aims:**

Recovery from substance use disorders (SUD) has traditionally been equated with abstinence. “Personal recovery” however emphasizes recovery as a unique and personal process, supported by changes in connectedness, hope, identity, meaning and empowerment. This study aimed to examine personal recovery in people receiving extended-release naltrexone (XR-NTX); specifically investigate changes in personal recovery during treatment, identify groups of participants following distinct trajectories of recovery, and characteristics predicting group-belonging.

**Methods:**

Overall change in recovery (Questionnaire about the Process of Recovery, QPR) score was assessed by linear mixed model in a subsample of 135 people with opioid use disorder (OUD) participating in a 24 + 28-week trial of XR-NTX. Growth mixture model was used to identify potential groups of people following distinct trajectories of personal recovery.

**Results:**

Overall, there was a significant change in QPR score during treatment. Four groups with distinct recovery trajectories were identified: “initially low– increase” (G1), “initially average– no change” (G2), “initially high– no change” (G3) and “initially high– increase” (G4). The groups were different with regards to level of psychological distress, social support, and the use of benzodiazepines. In addition, previous participation in opioid agonist treatment programs, current pain, life satisfaction, employment, heroin craving and previous use of heroin also differed between groups.

**Conclusions:**

Personal recovery among people receiving XR-NTX follows different trajectories, and various factors are associated with personal recovery. Particular attention regarding psychological distress, social support and heroin use among patients commencing XR-NTX treatment is important to facilitate successful recovery trajectories.

## Highlights

• People with OUD in XR-NTX treatment experienced an increase in personal recovery.
• Different recovery-trajectories were identified.
• Psychological distress, social support and heroin use at baseline predicted subsequent recovery trajectory.
• Patients with both initially poor and good outsets had much to gain in XR-NTX treatment, in terms of personal recovery.
• Findings offer important implications regarding the overall understanding of individual needs and required measures in XR-NTX treatment

## 1 Background

Recovery is a key concept in mental health and addiction services. In the field of substance use disorders (SUDs), the concept was traditionally synonymous with abstinence, for some (e.g. 12-step movements) meaning total abstinence from all substances. Nevertheless, it is now widely agreed that recovery can be supported by appropriate medications [1], and that abstinence, although important, is not the only prerequisite for recovery [2]. While an increased control over the intake of substances might be necessary (yet not sufficient) [3] or even pivotal [[4], [5], [6]] for further long term improvements, other factors, such as health and wellness [7], relationships [8] and improved quality of life [9] have been increasingly recognized as important for recovery.

In the mental health field, the concept of *personal recovery*, has gained wide acknowledgment as an alternative to the traditional focus on clinical recovery. The personal recovery concept is founded in service users’ experiences, and highlights the dynamic and multidimensional process of recovery as a unique, personal journey towards living a satisfying life, even with the limitations of the disorder [10]. Five core processes of personal recovery have been identified: Connectedness, Hope and optimism, Identity, Meaning and Empowerment, constituting the CHIME framework [11]. Studies examining addiction recovery, highlight recovery as a personal, ongoing process [5,12,13], emphasizing factors in line with the CHIME framework, such as support and relationships, identity and empowerment [5,[14], [15], [16]].

As the comorbidity between mental health problems and SUDs is high [17], the investigation of personal recovery in SUD settings, such as people with opioid use disorder (OUD), should also be relevant. Furthermore, personal recovery has been suggested as the bridging principle between mental health care and substance abuse treatment [18]. Personal recovery among people with SUD/OUD, or SUDs and mental health problems, has received little attention, and the few studies that exist are mostly qualitative. One such study examining personal recovery among members of Narcotics anonymous [19], found connectedness to be a crucial recovery-supportive element. When examined in the context of people with both SUD and mental health challenges (dual diagnosis; DD), recovery has been emphasized as a relational process [16]. A review from 2017 [20] showed the themes people with DD see as important for their personal recovery in large overlap with the themes identified in the CHIME framework.

Still, the notion of recovery from SUDs has been unclear, and remains somewhat ambiguous, despite increased interest in the concept. While there has been a progression towards including psychosocial outcomes and their association with long-term abstinence, the WHO still heavily emphasizes the abstinence aspect, defining recovery as “maintenance of abstinence from alcohol and/or other drug use by any means” [21]. Although multiple other definitions, stressing recovery as a process towards improvements in various life areas, exist, there is a tendency for researchers to sometimes implicitly define recovery in terms of substance use or abstinence, almost 15 years after Laudet's [5,12] call for emphasis on other factors and greater clarity of the term. This lack of consensus may also have contributed to the emphasis of abstinence in recovery [22].

OUD is a serious, potentially long-lasting condition with detrimental consequences for the individual and society as a whole. While the recommended treatment for OUD [23] is opioid agonist treatment (OAT), an alternative is the opioid antagonist extended-release naltrexone (XR-NTX) [24]. XR-NTX has shown good treatment outcomes when compared to OAT, such as a reduction in relapse rates, illicit drug use and depression or anxiety symptoms [[25], [26], [27]]. Treatment with XR-NTX involves blocking the effects of opioids, thus preventing the experience of pleasure or intoxication, and is associated with a decrease in opioid and substance use, as well as with psychosocial improvements [26,[28], [29], [30], [31], [32], [33]].

Previous studies have shown there are different subgroups of patients when it comes to opioid use patterns over time, both patients in OAT [34,35] and patients on XR-NTX [36]. Being a heterogeneous disorder [37], it can be expected that potential changes seen in the recovery process of people with OUD may not be representative for certain or all subgroups of patients. Identifying how individuals’ personal recovery process develops over time while receiving XR-NTX, as well as characteristics associated with different patterns might be important to achieve a better understanding of recovery among people with OUD receiving XR-NTX, and possibly informing timing or type of intervention efforts.

The main aim of this exploratory study was to examine the process of personal recovery among opioid dependent people receiving treatment with XR-NTX. Specifically, we sought to explore 1) possible changes in personal recovery during the course of treatment with XR-NTX; 2) whether there are groups of patients following distinct trajectories of personal recovery, and 3) if baseline characteristics could predict belonging to such groups with different QPR trajectories.

## 2 Materials and methods

### 2.1 Design

The present study is part of the Norwegian open-label, multi-center NaltRec study (“Long acting naltrexone for opioid addiction: the importance of mental, physical, and societal factors for sustained abstinence and recovery”). For further details on NaltRec, see Weimand et al. [38].

After complete detoxification from all opioids, an injectable suspension of 380 mg XR-NTX (Vivitrol®) was administered every 4th week for 24 weeks, with an optional 28 weeks treatment, giving a total of 52 weeks.

### 2.2 Setting and participants

The study was performed at five urban addiction clinics in Norway, in a naturalistic outpatient setting. Men and women, aged 18–65 years, with a diagnosis of opioid dependence according to DSM-IV criteria [39] were recruited from addiction clinics, detoxification wards or community health services. Participants had to be enrolled in an OAT program to ensure access to OAT if needed after ending participation. Treatment with XR-NTX was not available outside of the clinical trial.

Participants with severe psychiatric or somatic illness that could interfere with study participation, as well as pregnant or breastfeeding women, and people with a primary alcohol dependence were excluded.

### 2.3 Measures

#### 2.3.1 Personal recovery

Personal recovery was measured using the 15-item version of the *Questionnaire about the Process of Recovery* (QPR) [[40], [41], [42]]. QPR is one of the most widely used measures of personal recovery, has a strong evidence base and is related to the CHIME framework [43]. The items are rated on a 5-point scale from 0 = “disagree strongly” to 4 = “agree strongly”. The total sum score ranges from 0 to 60, with higher scores indicating higher degrees of personal recovery. Internal consistency, with a Cronbach's alpha of 0.93, test re-test reliability and convergent validity has been found to be high [40]. In this study, QPR was measured at baseline, 12, 24, 40 and 52 weeks.

#### 2.3.2 Covariates

Covariates included in this study were measured at baseline.

**Demographic variables** were measured using the Europ-ASI [44,45]. Number of close relationships was calculated based on a positive response in any of the 6 categories in questions H14-19, giving a maximum score of 6 if the respondent has close relationships in all categories.

Previous experiences of **traumatic events**, as well as a PTSD diagnosis was assessed using the MINI interview [46].

**Substance use** of alcohol, heroin, methadone or buprenorphine, other opioids, benzodiazepines, cocaine, amphetamines, cannabis and multiple substances, was measured using the Europ-ASI [44]. Use last 6 months was measured on a 5-point scale where 0 = “no use”, 1 = “sometimes but no more than 2–3 times a month”, 2 = “1–3 times a week”, 3 = “used daily, or almost daily”, 9 = “never used”. The scores were categorized into: no use (0 + 9), occasional use [38], frequent use (2 + 3). Historical severity of substance use was assessed as years of regular use [47].

**Craving** measured thoughts of substance use using an 11-point scale where participants were asked to indicate how often they had thought about “getting high on heroin” the last month, from 0 = “not at all” to 10 = “constantly/very much”.

**Current experience of pain** was measured using the single-item numeric pain rating scale [48,49], an 11-point scale where 0 = “no pain” and 10 = “worst pain imaginable”.

**Mental distress** was measured using the 25-item Hopkin's Symptom Checklist (H-SCL-25) [50] employing a 4-point scale ranging from 1 = “not at all” to 4 = “extremely.”

**Life satisfaction** was measured using the Temporal Satisfaction with Life Scale (TSWLS) [51]. Items are scored on a 7-point scale ranging from “strongly agree” to “strongly disagree”.

**Social** support was measured using the Interpersonal Support Evaluation List (ISEL-12) [52,53]. Items are scored on a 4-point scale ranging from 0 = “definitely false” to 3 = “definitely true.”

### 2.4 Statistical analysis

Baseline characteristics are presented as means and standard deviations (SDs) or frequencies and percentages. Due to pronounced skewness, medians, and min and max values were also included for substance use variables.

Overall change in QPR score in the entire sample was assessed by linear mixed model with random intercepts and fixed effects for time coded as dummy. As to exploratory approach, growth mixture model was estimated to identify possible unobserved groups of participants following distinct QPR trajectories [54]. The approach attempts to identify homogeneous groups of participants based on individual profiles by applying a set of statistical criteria. The criteria used were Bayes Information Criterion (BIC), where the smaller value implies better model, reasonable group sizes, and high average within-group probabilities. The models with up to five groups and third-order polynomial for trajectories were considered. BIC was applied to remove higher order terms in polynomials describing each trajectory and to reduce the number of groups. Model with smallest BIC value was chosen, and participants were assigned the class with highest posterior probability. The groups were further compared by ANOVA for continuous and Fisher-Freeman-Halton exact test for categorical baseline characteristics and substance use variables. Pairwise-comparisons were performed in post-hoc analyses. Due to relatively small group size and skewed distributions of some of the continuous variables, sensitivity analyses employing non-parametric tests were performed.

Cases receiving at least one XR-NTX injection but missing a QPR score at baseline were excluded. Missing values on single items lead to a missing overall score. Participants with and without missing values on QPR were compared by Fisher-Freeman-Halton exact- or independent samples *t*-test. Due to the exploratory nature of the study no adjustment for multiple testing was implemented. All analyses were conducted in STATA v16. All tests were two-sided and results with p-values below 0.05 were considered statistically significant.

### 2.5 Ethics

The study was approved by the Regional Committee for Medical and Health Research Ethics Southeast Norway, the Norwegian Medicines Agency, and the boards of research ethics at the participating hospitals, and conducted in accordance with the ethical principles of the Declaration of Helsinki [55]. Patient data was handled according to the General Data Protection Regulation (GDPR), and National Personal Data protection regulations. The study is registered at clinicaltrials.gov (NCT01717963)*.*

## 3 Results

### 3.1 Participant characteristics

The overall NaltRec study included 162 persons, of whom 138 received at least one injection of XR-NTX. The sample for the present study consisted of the 135 participants who received at least one injection, and in addition filled out the QPR at baseline. The sociodemographic variables of the sample are presented in Table 1.

**Table 1:** Baseline characteristics, N = 135.

Characteristic	Statistic
Gender, female, n (%)	30 (22.2)
Age, mean (SD)	37.6 (9.4)
In OAT before enrolment in study, Yes, n (%)	81 (60.0)
No. of XR-NTX injections received	
mean (SD)	9.6 (5.2)
median (min-max)	11.0 (2.0–15.0)
Years of completed education, mean (SD)	12.0 (2.4)
Working last 4 weeks, n (%)	30 (22.2)
Common housing arrangement past 3 years, n (%)	
Alone	80 (59.3)
With family or friends	47 (34.8)
In prison/institution	4 (3.0)
No stable living situation	4 (3.0)
Spends most of free/leisure time with …, n (%)	
Family or friends without problematic substance use	65 (48.1)
Family or friends with problematic substance use	18 (13.3)
Alone	52 (38.5)
Years of regular heroin use	
mean (SD)*	6.3 (6.1)
median (min-max)	5.0 (0.0–38.0)
Years of regular use of methadone/buprenorphine	
mean (SD)	5.3 (5.0)
median (min-max)	4.0 (0.0–20.0)
Years of regular benzodiazepine use	
mean (SD)	6.3 (7.6)
median (min-max)	4.0 (0.0–36.0)
No. of days used last 4 weeks	
Heroin	
mean (SD)	6.8 (10.2)
median (min-max)	0.0 (0.0–28.0)
Methadone/buprenorphine	
mean (SD)	16.8 (12.4)
median (min-max)	25.5 (0.0–28.0)
Benzodiazepines	
mean (SD)	10.0 (11.6)
median (min-max)	3.0 (0.0–28.0)
Exposure to traumatic event, n (%)**	111 (84.1)
Fills MINI-criteria for PTSD, n (%)**	20 (15.2)

*2 missing, **3 missing.

### 3.2 QPR score over time

**Fig. 1:**
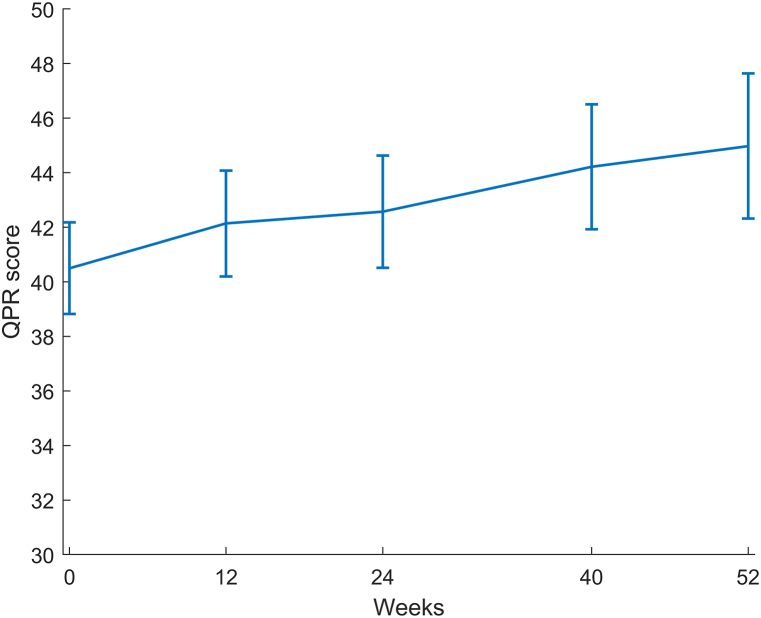
QPR score over time; the values are mean QPR scores estimated from linear mixed model, while bars represent the 95% confidence interval.

Descriptive statistics of QPR score at each time point are presented in Table 2. Compared to participants without missing QPR values, those with missing values were more frequently women, more often not at work the last 4 weeks, fewer were satisfied with how their spare time was spent, were slightly more often unemployed, and more used methadone/buprenorphine frequently (results not shown). During the course of treatment, the observed mean QPR score for the group as a whole increased by 7 points. According to linear mixed model, taking within-participant correlations into account, this increase was estimated to be approximately 4.5 points. QPR score increased significantly from baseline to week 24, 40 and 52 - but not to week 12 (Table 2 and Fig. 1). No other changes in QPR score were significant.

**Table 2:** Descriptive statistics of outcome variable and results of linear mixed model (RC = regression coefficient, CI = confidence interval).

	n	Mean (SD)	RC (95% CI)	*p-value*
Baseline – ref.	135	40.5 (10.3)	0	
Week 12	92	42.3 (9.9)	1.64 (−0.26; 3.55)	*0.091*
Week 24	78	43.1 (10.5)	2.07 (0.05; 4.10)	***0.045***
Week 40	58	45.2 (8.0)	3.72 (1.46; 5.98)	***0.001***
Week 52	39	47.5 (8.6)	4.48 (1.85; 7.11)	***0.001***

### 3.3 Personal recovery trajectories

**Fig. 2:**
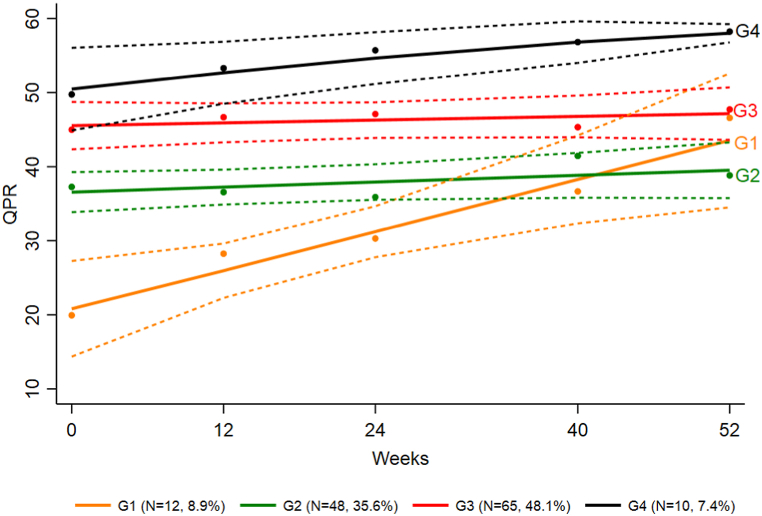
Results of growth mixture model (should include color)

A growth mixture model identified four groups of participants following distinct QPR trajectories, coined the “initially low– increase” (G1), “initially average– no change” (G2), “initially high– no change” (G3) and “initially high– increase” (G4) (Table 3, Fig. 2).

**Table 3:** Results of growth mixture model (RC = regression coefficient).

Parameter	G1 (N = 12, 8.9%)		G2 (N = 48, 35.6%)		G3 (N = 65, 48.1%)		G4 (N = 10, 7.4%)	
	RC (SE)	*p-value*	RC (SE)	*p-value*	RC (SE)	*p-value*	RC (SE)	*p-value*
Intercept	20.64 (2.50)	***<0.001***	36.57 (1.36)	***<0.001***	45.58 (1.39)	***<0.001***	50.76 (2.12)	***<0.001***
Linear	0.44 (0.11)	***<0.001***	0.06 (0.04)	*0.171*	0.03 (0.04)	*0.407*	0.21 (0.08)	***0.007***
Av.prob.^a^	0.88		0.80		0.75		0.90	

a Average within-group probability.

High average within-group probabilities indicated that the groups were homogenous, with only a few participants having nearly equal (around 0.5) posterior probabilities across the groups (one in G1 and G2, three in G2 and G3, and one in G3 and G4). The entropy for the final model was estimated to be 0.65. The baseline values of QPR were significantly different between groups, according to non-overlapping 95% CIs at baseline. Even though the two “improvement” groups were small, they were quite distinct and exhibited a significant linear change.

G2 and G3 constituted the majority of participants (35.6% and 48.1%, respectively), and showed no significant change in score over the course of treatment.

G1 had the lowest starting point, with initial QPR scores almost half of those of G2. However, G1 exhibited a significant change in score during the course of treatment, ending up with a QPR score higher than G2, and closer to that of G3 at 52 weeks.

G4 had the highest starting point, about 30 points higher than G1, and still exhibited a significant increase in score.

### 3.4 Covariates associated with change in QPR

Table 4 and Table 5 present comparison of groups with respect to baseline characteristics and substance use variables, respectively. Sensitivity analysis showed no major deviations from reported results.

**Table 4:**
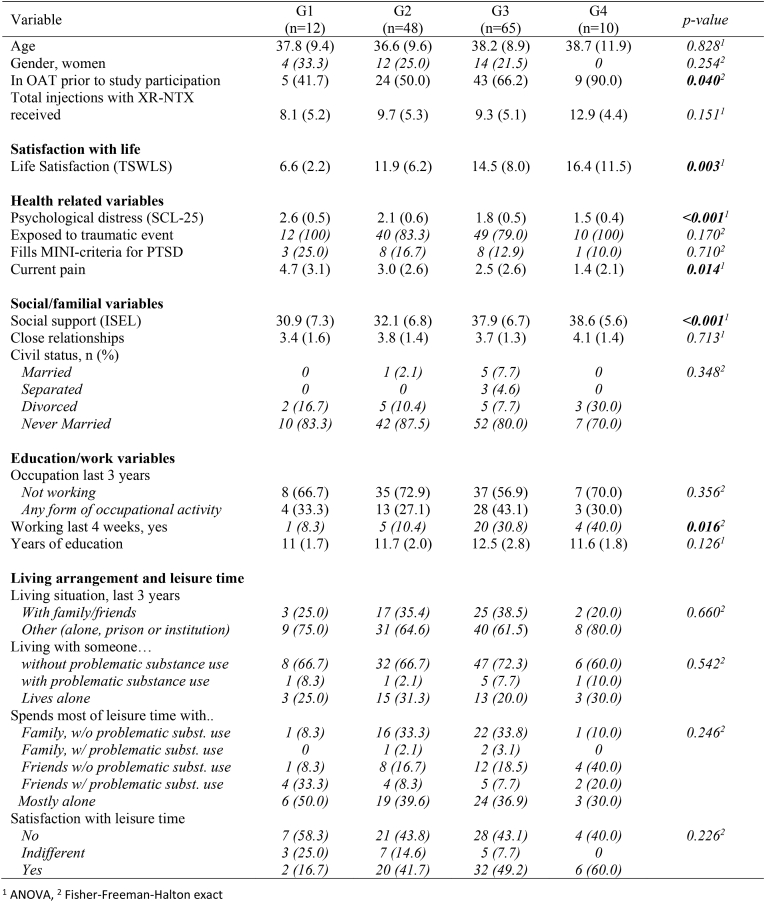
Descriptive statistics within groups (N = 135), presented as mean (SD) or *N(%) [italic]*.

**Table 5:**
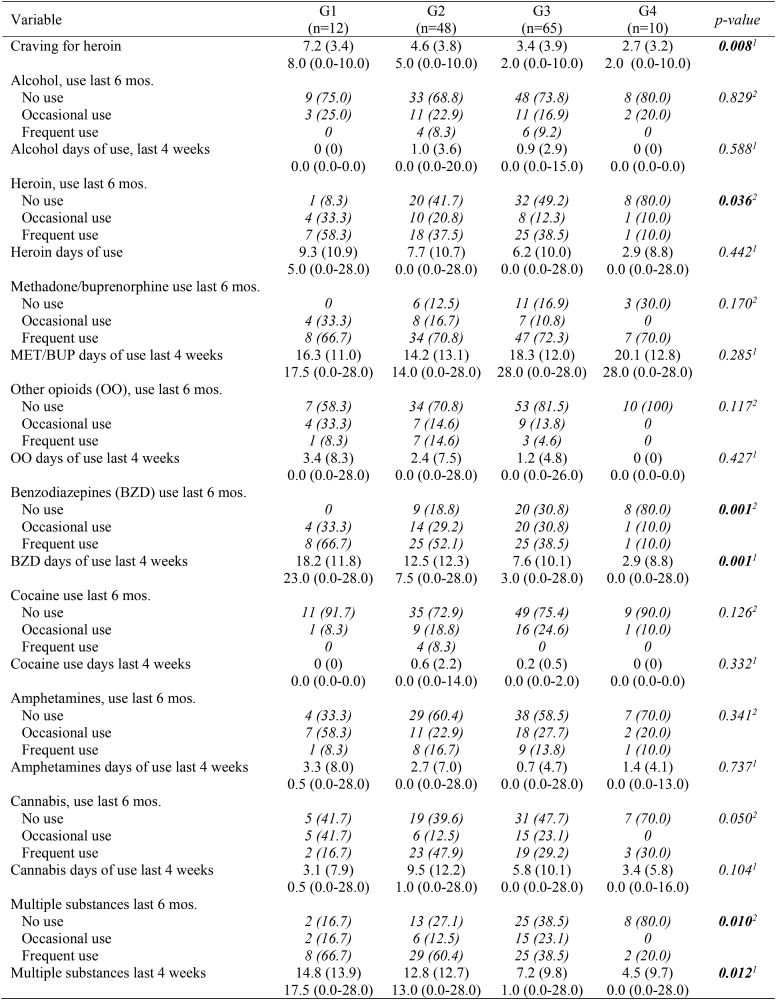
Substance use variables, presented as mean (SD), median (min-max) or *N(%) [Italic]*.

No significant differences between groups were found for age or gender, or sociodemographic variables. However, the groups differed significantly regarding OAT status at the time of study enrolment; in G1 41.7% were in OAT, whereas this number was 90% in G4.

Post hoc tests (Table 6) showed G1 differed significantly from other groups with respect to most variables where the overall significant difference between groups was found, except for the variable “working last 4 weeks”, where no differences between G1 and other groups were found.

**Table 6:** Results of post-hoc analyses. Pairwise comparisons between groups. Numbers are p-values (only significant differences presented).

Variable	G1 vs G2	G1 vs G3	G1 vs G4	G2 vs G3	G2 vs G4	G3 vs G4
In OAT prior to study participation			*0.031*		*0.033*	
Psychological distress (SCL-25)	*0.006*	*<0.001*	*<0.001*	*0.003*	*0.002*	
Social support (ISEL)		*0.002*	*0.013*	*<0.001*	*0.006*	
Current pain		*0.006*	*0.011*			
Working last 4 weeks				*0.012*	*0.039*	
Life Satisfaction (TSWLS)	*0.005*	*0.001*	*0.009*			
Craving for heroin	*0.040*	*0.003*	*0.005*			
Heroin, use last 6 mos, n (%)		*0.012*	*0.002*			
BZD use last 6 mos		*0.040*	*<0.001*		*0.001*	*0.011*
BZD days of use last 4 weeks		*0.002*	*0.003*	*0.022*	*0.023*	
Multiple substances last 6 mos			*0.014*		*0.010*	
Multiple substances last 4 weeks			*0.023*	*0.010*		

G1 showed significantly higher scores on overall mental distress and lower perceived social support than the other groups, and experienced higher pain and lower life satisfaction than G3 and G4. This was also the case for the craving variable.

In G2 the social support as well as the mental distress scores were significantly higher than in both G3 and G4. There were however no differences in how participants or the interviewer rated their need for help with psychological/emotional problems, or regarding experience of traumatic events or PTSD diagnosis. Also, G2 was significantly less likely to be working in the last 4 weeks than G3 or G4.

For the substance use variables, years of regular use did not differ between groups for any substance. No differences between groups were found on use of alcohol, other opioids, cocaine, amphetamines, or cannabis. For heroin, use during the last 6 months, but use the last 4 weeks, differed between groups. For benzodiazepines, G4 had a significantly lower use than the other groups, both during the last 4 weeks and 6 months.

## 4 Discussion

The identification of differential trajectories for personal recovery adds understanding to the recovery process during the course of XR-NTX treatment, highlighting different subgroups of patients which may benefit differently from XR-NTX in terms of personal recovery. In the following, we will shortly discuss the change in QPR score in the course of treatment, then focus mainly on the groups of patients following distinct QPR trajectories and associated characteristics.

### 4.1 1: Personal recovery during the course of treatment

There was an overall increase in personal recovery from baseline to 24, 40 and 52 weeks. Previous findings have shown people receiving XR-NTX exhibit higher life satisfaction [56], which is closely related to quality of life (QoL) [57,58]. QoL has been shown to be significantly associated with personal recovery, indicating that constructs of both QoL and personal recovery include a personal process strongly associated with perceived empowerment [59]. In line with viewing recovery as a process, the change was not significant from baseline to 12 weeks, supporting the notion that the process requires a certain period of time.

While a concern might be the QPR is not previously used in SUD settings, the processes important in personal recovery (Connectedness, Hope, Identity, Meaning and Empowerment) are easily seen as universal, nonspecific processes important for any recovery. Our findings add a preliminary support to the use of the QPR in SUD populations, provided further examination of the concept and validation of the measure.

### 4.2 2: Trajectories of personal recovery

The identification of four groups of patients following distinct recovery trajectories provides an increased understanding of personal recovery among patients receiving XR-NTX in this Norwegian study. Most patients (i.e. the two “no-change groups”, constituting almost 84%) reported little change in QPR score. This might not be surprising, considering SUD-recovery can be a lengthy, ongoing process [12,60,61]. In addition, it is suggested the QPR should not only be used to give a total recovery score, but also to facilitate engagement and individual goalsetting [62]. Thus it would possibly be more meaningful for some patients to look at subdomains of recovery related to individual goals, instead of the total QPR score.

The two “improvement” groups (G1 and G4), although small, represent notable patterns of recovery. Firstly, G4 with considerably high QPR scores at baseline, both relative to the other groups and to previous findings [42,63], experienced a significant increase in QPR during the course of treatment. This is in contrast with the notion of ceiling effects [64], where one would expect patients scoring initially high to experience limited increase. Possibly this group of patients is especially “high-functioning”, and already in the process of recovery, a hypothesis strengthened by the higher OAT participation, and the lower burden of pain and psychological distress, compared to the other groups. XR-NTX treatment may thus have propelled their recovery process, in line with qualitative findings that many patients seeking XR-NTX treatment do so to advance their recovery by exiting OAT [65,66].

Secondly, the initially low scoring G1 seems to represent a group of patients with likely much to gain by commencing XR-NTX treatment, as they surpassed G2 in QPR score, and also had the largest increase in score during the course of treatment. Nevertheless, they are also a group of patients experiencing considerable burden, and clinicians should be aware of the serious struggle these patients face when entering treatment. Factors differentiating between trajectories further underline the importance of “something” in addition to « just the injection» in personal recovery among people receiving XR-NTX.

### 4.3 Characteristics associated with recovery trajectories

Several pre-treatment characteristics were associated with QPR trajectory, and thus significant for personal recovery in people receiving XR-NTX. Demographic characteristics did however not vary by group, underlining personal recovery as a universal process, not tied to differences in age, gender or education. Nevertheless, individual factors, such as pattern of previous heroin use, psychological distress or social support did impact recovery trajectory. This is in line with findings of social support [67], or severity of mental problems [68] as important covariates of personal recovery.

The initially low scoring G1 was more likely to not be in OAT, experience higher psychological distress, more pain, lower life satisfaction and social support, and exhibit more heroin and benzodiazepine use, implying a lower functioning in this group. Although we do not know how the associated characteristics developed over the course of treatment, pain and psychological distress have not been found to rise during XR-NTX treatment [27,69], while life satisfaction likely increases [56].

In SUD recovery, substance use, and its discontinuation, has been considered important, and in this study, heroin use in the last 6 months emerged as a factor distinguishing between groups. However, heroin use last 4 weeks did not differ significantly, and neither did any substance use, other than of benzodiazepines and multiple substances. The lack of difference in use of heroin in the last month might however be due to participants overall having made the decision to enter XR-NTX treatment, and thus reducing their use to a similar level.

Regarding psychological distress, except for G4, participants had H-SCL scores above the cut-off for clinically significant distress. However we found no differences between groups on previous experiences of traumatic events or PTSD diagnosis, which may be surprising given that previous experiences of trauma or abuse are associated with OUD [70], and persistent opioid use [71]. Co-occurring mental disorders are common among people with SUD [17] and contribute negatively to the course and treatment of OUD [72], while addressing mental problems likely enhances long-term abstinence [71]. Mental distress has been associated with higher levels of impulsivity, hyperactivity and inattention among patients seeking treatment with XR-NTX, and is highlighted as important to focus on when considering treatment options for OUD [73]. Further, there are indications of a reduction in mental symptoms during XR-NTX-treatment [27]. Although our analysis does not allow for a longitudinal examination of associated factors, one hypothesis regarding mental distress could be that the increase in personal recovery may be connected to a decrease in mental distress, in line with qualitative findings [66] that mental health difficulties are important for how well the opioid blockade in XR-NTX treatment is handled, or tolerated. For instance, people with underlying mental health challenges, or who experience great emotional or mental distress may find it especially challenging to not be able to escape into opioid intoxication when struggling.

Social support is related to the concept of connectedness, and has been shown to be important in SUD recovery [19,61]. In the present study, social support at baseline differed significantly between groups of high vs. low baseline QPR; G1 and G2 with the lower baseline QPR score differed from G3 and G4, but not from each other. Thus a lower social support score at baseline would mean a lower baseline QPR score (G1/G2), regardless of whether an increase in QPR score was experienced later in treatment (G1/G4) or not (G2/G3). Nevertheless, all groups were above the midpoint on the ISEL, indicating positive views of the social support available. Interestingly, no other social or relational variables such as number of close relationships differed significantly between groups.

On a final note, we do not know if treatment increased mental wellbeing, social support, or some other factor, thereby increasing QPR score. However, the factors found to predict QPR trajectory are nevertheless relevant to recognize and observe when patients commence XR-NTX treatment.

### 4.4 Strengths and Limitations

The current study had several strengths. The study is naturalistic, includes a relatively large sample size and explores personal recovery, both over time, and in a novel context. While it should be kept in mind the study is exploratory in nature, providing patterns that may be worth examining further, some limitations should especially be noted.

Notably, the longitudinal, observational design, although allowing for an examination of personal recovery over time, does not allow for causal inferences to be made. Further, the study offers an assessment of associations between baseline characteristics and QPR score, and subsequent trajectory, not allowing for comparisons between groups on different factors over the course of treatment. The baseline characteristics were possibly influenced by the patients’ current situation, e.g. withdrawal or excitement to soon start a new treatment. There was a notable dropout of participants, resulting in missing values in the outcome variable (QPR) at later time points, which may have caused bias in the results. Another limitation lies in two of the identified groups being rather small. The QPR has not been previously used nor validated in a SUD setting, nor in the context of XR-NTX treatment, and is commonly used in the context of recovery from serious mental illness. Although the concept of personal recovery should be applicable, it cannot be certain that the use of the QPR is suitable for this population, type of study, or covers all areas of recovery relevant among people with SUD. For example, abstinence or reduction in substance use is commonly seen as an important factor in recovery from SUD [74], but it is not part of the CHIME-model. Thus QPR, may not capture all major aspects of recovery for such a population.

Personal recovery has not previously been examined in the context of XR-NTX treatment, and this study offers an important addition to the understanding of both recovery and XR-NTX treatment. The findings point to several important focus areas for clinicians. For instance, several pretreatment variables (such as mental distress, social support, use of benzodiazepines or opioid craving) are highlighted as associated with subsequent recovery. As such, it may be useful for clinicians to assess these variables in patients considering commencing XR-NTX treatment. Further, clinical awareness and consideration of these factors can be important both when assessing patients interested in XR-NTX treatment and in the evaluation and facilitation of successful treatment and recovery pathways for patients in treatment. As such, the results offer important insights to policy makers, underlining that different groups of patients can benefit from treatment, also patients in different stages of recovery. At the same time results show that certain requisites will perhaps need to be present for a given patient to benefit. The study highlights the heterogeneity in people with OUD, and underlines the individual and diverse trajectories of recovery.

## 5 Conclusions

People with OUD in XR-NTX treatment experienced an increase in personal recovery. Especially patients with an initially poor outset in terms of psychological distress, social support and heroin use might have much to gain by commencing XR-NTX in terms of recovery, as well as those already in an active recovery process. More research is needed to further explore why some individuals seem to benefit more than others regarding personal recovery during treatment. The findings offer important clinical implications regarding the overall understanding of individual needs and required measures in clinical treatment, as well as may contribute to helping more patients benefit from XR-NTX treatment. Findings further underline the importance of policy makers to provide treatments according to the patients’ needs.

## Ethics approval and consent to participate

Ethical approval for the NaltRec trial, including the present study, was granted by the Norwegian Regional Ethical Committees for Medical and 10.13039/100005622Health Research Ethics (REK) committee South East A (# 2018/132), by the personal data protection representative for each of the sites, and by the Norwegian Medicine Agency (NOMA), EudraCT Code 2017-004706-18. The trial is registered at Clinicaltrials.gov # NCT03647774. It was first registered on Aug 28, 2018, before first participant inclusion on Sep 21, 2018 [38]. All participants gave written, informed consent for their participation.

## Consent for publication

All participants gave general written, informed consent for publication.

## Availability of data and material

The data used in this study are based on a still ongoing study that will be finalized in 2025. According to current Norwegian regulations and practice, the data will then be anonymized and deposited in a publicly available data repository (e.g., The Norwegian Center for Research Data).

## Funding

This work was supported by unrestricted grants from the 10.13039/501100005416Research Council of Norway (grant 269,864) and the 10.13039/501100006095South-Eastern Norway Regional Health Authority (2,019,105). Extended-release naltrexone was provided by Alkermes at no cost in accordance with an Investigator Initiated Trial agreement. Participating sites contributed with study personnel.

## Author contribution statement

Anne Marciuch: Performed the experiments; Analyzed and interpreted the data; Wrote the paper. Bente Birkeland: Analyzed and interpreted the data; Wrote the paper. Jūratė Šaltytė Benth: Analyzed and interpreted the data; Wrote the paper. Kristin Klemmetsby Solli: Performed the experiments; Wrote the paper. Lars Tanum: Conceived and designed the experiments; Performed the experiments; Wrote the paper. Ida Mathisen: Performed the experiments. Bente Weimand: Conceived and designed the experiments; Performed the experiments; Analyzed and interpreted the data; Wrote the paper.

## Data availability statement

Data will be made available on request.

## Declaration of competing interest

The authors declare that they have no known competing financial interests or personal relationships that could have appeared to influence the work reported in this paper.
